# THE RELATIONSHIPS OF VOICE PROSODY MEASURES TO COGNITIVE CHANGES OVER 10 YEARS

**DOI:** 10.1093/geroni/igad104.3096

**Published:** 2023-12-21

**Authors:** Elizabeth Mahon, Margie Lachman

**Affiliations:** Brandeis University, Waltham, Massachusetts, United States; Brandeis University, Waltham, Massachusetts, United States

## Abstract

Voice prosody measures, especially jitter and shimmer, can differentiate cognitive impairment from healthy cognition, and may be promising early biomarkers for identifying high risk before later-life disease onset. In our past work, higher jitter and lower pulse assessed during the second cognitive assessment were associated with 10-year cognitive declines in middle-age and older adults from the Midlife in the U.S. (MIDUS) study. The current study assessed voice biomarkers taken at baseline, 10 years before the second cognitive assessment, and measured voice on speech that was independent of the cognitive assessments to address the possible influence of cognitive performance on concurrent voice prosody measures. Participants (ages 34 to 82 at the first occasion) were included if they were in the Boston Longitudinal Study (BOLOS, a subset of MIDUS) and had voice measures collected from the audio recordings of the cognitive interviews. Multilevel analyses controlled for age, sex, education, race, neurological conditions, and depressive symptoms at baseline. Higher jitter, lower shimmer, and lower pulse predicted greater 10-year declines in episodic memory. Higher pitch and lower pulse were associated with greater increases in reaction time on an executive functioning task. Thus, voice biomarkers previously associated with cognitive impairment could be useful as early indicators of later-life cognitive declines. Identifying voice biomarkers that could predict 10-year cognitive change may facilitate earlier intervention for decline through modification of disease risk factors.

